# The choroidal nervous system: a link between mineralocorticoid receptor and pachychoroid

**DOI:** 10.1007/s00401-023-02628-3

**Published:** 2023-09-08

**Authors:** Bastien Leclercq, Allon Weiner, Marta Zola, Dan Mejlacowicz, Patricia Lassiaz, Laurent Jonet, Emmanuelle Gélizé, Julie Perrot, Say Viengchareun, Min Zhao, Francine Behar-Cohen

**Affiliations:** 1 Centre de Recherche des Cordeliers, Inserm, Université Paris Cité, Sorbonne Université, Physiopathology of Ocular Diseases: Therapeutic Innovations, 15 rue de l’Ecole de Médecine, 75006 Paris, France; 2 Sorbonne Université, Inserm, Centre d’Immunologie et des Maladies Infectieuses, Cimi-Paris, Paris, France; 3 Ophthalmopole Cochin University Hospital, Assistance Publique-Hôpitaux de Paris, Paris, France; 4 Hopital Foch, Suresnes, France; 5 Université Paris-Saclay, Inserm, Physiologie et Physiopathologie Endocriniennes, 94276 Le Kremlin-Bicêtre, France

**Keywords:** Mineralocorticoid, Pachychoroid, Neuropathy, Choroid, Innervation, CSCR

## Abstract

Central serous chorioretinopathy (CSCR) belongs to the pachychoroid spectrum, a pathological phenotype of the choroidal vasculature, in which blood flow is under the choroidal nervous system (ChNS) regulation. The pathogenesis of CSCR is multifactorial, with the most recognised risk factor being intake of glucocorticoids, which activate both the gluco- and the mineralocorticoid (MR) receptors. As MR over-activation is pathogenic in the retina and choroid, it could mediate the pathogenic effects of glucocorticoids in CSCR. But the role of MR signalling in pachychoroid is unknown and whether it affects the ChNS has not been explored. Using anatomo-neurochemical characterisation of the ChNS in rodents and humans, we discovered that beside innervation of arteries, choroidal veins and choriocapillaris are also innervated, suggesting that the entire choroidal vasculature is under neural control. The numerous synapses together with calcitonin gene-related peptide (CGRP) vesicles juxtaposed to choroidal macrophages indicate a neuro-immune crosstalk. Using ultrastructural approaches, we show that transgenic mice overexpressing human MR, display a pachychoroid-like phenotype, with signs of choroidal neuropathy including myelin abnormalities, accumulation and enlargement of mitochondria and nerves vacuolization. Transcriptomic analysis of the RPE/choroid complex in the transgenic mice reveals regulation of corticoids target genes, known to intervene in nerve pathophysiology, such as *Lcn2*, *rdas1/dexras1, S100a8 and S100a9*, rabphilin 3a (*Rph3a*), secretogranin (*Scg2*) and Kinesin Family Member 5A (*Kif5a*). Genes belonging to pathways related to vasculature development, hypoxia, epithelial cell apoptosis, epithelial mesenchymal transition, and inflammation, support the pachychoroid phenotype and highlight downstream molecular targets. Hypotheses on the imaging phenotype of pachychoroid in humans are put forward in the light of these new data. Our results provide evidence that MR overactivation causes a choroidal neuropathy that could explain the pachychoroid phenotype found in transgenic mice overexpressing human MR. In patients with pachychoroid and CSCR in which systemic dysautonomia has been demonstrated, MR-induced choroidal neuropathy could be the missing link between corticoids and pachychoroid.

**Supplementary Information:**

The online version contains supplementary material available at 10.1007/s00401-023-02628-3.

## Introduction

The choroid is a highly vascularized structure located between the sclera and the retinal pigment epithelium (RPE), responsible for nutriment and oxygen supply to the photoreceptor cells that ensure phototransduction. In humans, the suprachoroid, leaning against the sclera, is followed by three vascular layers with vessels of decreasing size, Haller's layer, Sattler's layer and then the choriocapillaris layer, which is juxtaposed to Bruch's membrane overhung by the RPE [33] (Supplementary Fig. 1). The choroid ensures several functions essential for retinal homeostasis. It meets the metabolic needs of photoreceptors through adjusted blood perfusion, controls the oncotic gradients between the retina and the sclera, ensures heat dissipation and contributes to focalization by thickness variations and by non-vascular smooth muscles activity [60, 75]. Choroidal and RPE dysfunction together with choroidal inflammation, are the primary mechanisms of numerous eye diseases.

Pachychoroid is an imaging phenotype, identified on spectral domain optical coherence tomography (SD-OCT) by the presence of dilated choroidal veins, possibly resulting from venous overload [66], and by an effacement of the overlying capillaries. Indocyanine green (ICG) angiography shows vascular hyper-permeability and typical mid-phase hyper-fluorescent plaques that correspond to focal area of abnormal RPE [10, 30]. The occurrence of RPE leaks may cause serous retinal detachments that characterise Central serous chorioretinopathy (CSCR), a well-defined entity [4, 10, 11, 25, 38] within the “pachychoroid disease spectrum” [9, 19]. CSCR is the fourth cause of visual impairment in mid-aged men and can cause permanent loss of vision in complex cases [20, 24, 54]. The pathogenesis of CSCR is multifactorial and not fully understood with glucocorticoids (GCs) intake being the most unanimously recognised risk factor [12, 23, 56], through mechanisms that remain unclear. Indeed, GCs are known as potent vasoconstrictor and anti-edematous drugs [77, 83] but in CSCR that is induced and aggravated by GCs, choroidal vessels are vasodilated and oedema results from sub-retinal fluid accumulation. How GCs favour vascular dilation and oedema in CSCR remains to be understood.

GCs exert their effects via two receptors, the glucocorticoid receptor (GR) and the mineralocorticoid receptor (MR). Whilst glucocorticoids action via GR activation is anti-inflammatory, MR pathway activation has been associated with deleterious effects, such as endothelial dysfunction and vascular inflammation, oxidative stress, inflammation and fibrosis in a number of organs [42]. In the retina, MR pathway over-activation has been shown to be pro-inflammatory, pro-edematous and pro-angiogenic [5, 78, 79]. Hyper-activation of MR pathway has thus been proposed as a potential mechanism explaining the deleterious effects of GCs in CSCR [24].

More recently, it has been reported that mouse over-expressing human MR display choroidal and RPE pathology including choroidal vessels dilation, increased permeability and RPE disorganisation, mimicking pachychoroid epitheliopathy [15, 76]. On the other hand, transcriptomics analysis of the RPE/choroid complex performed after aldosterone intraocular injection, which activates MR, revealed a significant down-regulation of pathways associated with neuronal activity, synapse organisation, neurotransmitter/vesicles transports and exocytosis [15] suggesting a dysregulation of the choroidal nervous system (ChNS). But to date, the role of corticoids on the ChNS has not been explored.

In the past 40 years, several groups have described the ChNS in birds and mammals, showing sensory input from the trigeminal ganglion (TG), sympathetic input from the superior cervical ganglion (SCG) and a parasympathetic input from the pterygopalatine/sphenopalatine ganglion (PPG), that forms a dense and complex network [57, 62], that controls all choroid functions. The sympathetic input is supplying the choroid with noradrenaline (NA) and neuropeptide Y (NPY) mediating a vasoconstrictive effect whilst decreasing the choroidal blood flow (ChBF) to limit over-perfusion in case of elevated systemic blood pressure [8, 62]. The parasympathetic input mediated by acetylcholine (Ach), nitrite oxide synthase (NOS) and vasoactive intestinal peptide (VIP), induces vasodilation and increases the ChBF to compensate systemic hypotension [44, 62]. The sensory fibres releasing calcitonin gene-related peptide (CGRP) and substance P (SP), might induce vasodilatation in response to temperature raise [6, 62, 68]. In humans, intrinsic choroidal neurons (IChNS) have also been described, organised in plexus and concentrated below the fovea in the deep choroidal layers [32, 61, 62]. They are mainly NOS and VIP-positive and some are CGRP-positive [27], indicating parasympathetic/sensory vasodilatory effect. They also receive sympathetic (tyrosine hydroxylase (TH)-positive), parasympathetic (choline acetyltransferase (ChAT)-positive) and sensory terminals (CGRP-positive), suggesting that autonomous and sensory regulations [53] contribute to foveal ChBF control and to the retinal alignment within the focal plan [57, 62]. Overall, the ChNS can regulate the ChBF upon sensory responsiveness to pressure and temperature variations. But whether corticoids intervene in the ChNS regulations have not been evaluated.

Based on our previous observation that MR overexpression causes a pachychoroid-like phenotype in mice and that over-activation of MR by aldosterone regulates mainly the expression of genes involved in ChNS components [15], we hypothesise that MR-induced pachychoroid-like phenotype could result from a choroidal neuropathy.

## Materials and methods

### Human tissue preparation

Two eyes from two donors (for science) were obtained from the Lausanne Eye Bank (CER-VD N°340/15). The individuals were not diabetic and had no specific ocular disease history, but the retina imaging of these donors was not accessible. One eye, from a 71-year-old male donor (postmortem delay of 15 h) was fixed in 4% paraformaldehyde (PFA) overnight and preserved in 1% PFA for flat-mount immunohistochemistry. After removing the cornea and lens, the remaining posterior segment of the eye was flat-mounted and dissected to remove the neural retina. The posterior segment was cut into 4 quarters and the RPE/choroid was separated from the sclera after section of the vortex veins, the RPE cells were removed manually using a soft sponge. Immunohistochemistry was performed on the remaining choroid containing minimal RPE cells on the surface, using the procedures and antibodies described in the “Immunostaining” section. The other eye, from a 69-year-old woman (postmortem delay of 10 h) was processed for semi-thin and ultrathin sections. The eye was fixed in 2.5% glutaraldehyde in cacodylate buffer (0.1 M, pH 7.4). After one hour fixation, it was dissected, and the anterior segment and lens were removed. The posterior part was fixed for a further 5 h, dehydrated in a graded alcohol series (50%, 70%, 95% and 100%), cut is several pieces and embedded in epoxy resin. Semi-thin sections (1 µm) were cut and stained with toluidine blue. Ultrathin sections (80 nm) were contrasted by uranyl acetate and lead citrate and observed with a transmission electron microscope and photographed.

### Animals and ethical concerns

Wild-type male and female Sprague Dawley rats (*n* = 30) were obtained from Janvier Labs (Le Genest-Saint-Isle, France) and kept in a 12:12 light dark cycle with drink/food ad-libitum at the Function Exploration Center (CEF, Campus des Cordeliers, Université Paris-Sorbonne, Paris). Sprague Dawley rats were used to avoid the non-specific staining of monoclonal mouse antibodies and to limit the interference of choroidal pigmentation. To study the consequences of MR overexpression on the ChNS, we used P1.hMR mice, which overexpress the human MR (hMR) gene, under the control of the proximal P1 promoter of the *NR3C2* in all aldosterone target tissues. P1.hMR mice, generated under a C57Bl/6 background, were generously shared by Dr Say Viengchareun (Inserm U1185, Paris-Saclay University, le Kremlin Bicêtre, France). This model gave new insights into MR-related pathophysiology in cardiac and renal functions [48]. Experiments have been conducted in accordance with the European Communities Council Directive 86/609/EEC and the protocols have been approved by local ethical committees (#25158-2020041903191320 v3).

### Triamcinolone treatment in P1.hMR mice and histological analysis

Females & Males P1.hMR mice (*n* = 5) and their littermates (*n* = 5) received a single sub-conjunctival injection of Triamcinolone (1 mg/kg). One week after injection, mice were euthanized by cervical dislocation and eyes were collected and fixed for 2 h in a mix of 4% paraformaldehyde and 0.5% glutaraldehyde. Samples were dehydrated in a graded alcohol series (70%, 95%) for 2 h each before being embedded using HistoResin standard kit protocol (BioSystems, Hofackerstrasse, Switzerland). Then, 5 µm transversal eye sections were performed using a microtome, dried on slides, and stained for 2 min in 1% Toluidine blue. Finally, sections were observed in bright-field microscopy with oil immersion (model Olympus BX51, Olympus, Rungis, France).

### Sampling and rat tissue preparation for immunostaining

Male and female rats were euthanized by intraperitoneal injection of EUTHASOL VET (300 mg/Kg). After death, eyes were removed and incubated in 4% PFA during 1 h for fixation. Then, eyes were either dissected for flat-mounting of the sclera–choroid–RPE complex or incubated in 30% sucrose solution over-night before being embedded in Tissue-Tek OCT (Optimal cutting temperature compound) and stored at − 80°C. The sclera–choroid–RPE complexes were immediately used for immunostaining. Transverse cryosections of 12µm were performed using a cryostat, mounted on superfrost plus slides (Epredia™ SuperFrost Plus™, Fisher Scientific, Illkirch, France) and stored at -20°C before immunostaining protocol.

### Immunostaining

Rat sclera–choroidal–RPE complexes and human choroid pieces were incubated for 1 h in a pre-incubation solution (phosphate-buffered saline (PBS) 0.1M, 10% normal goat serum, 0.01% triton X100) at room temperature (RT). Then, tissues were incubated with the primary antibodies at appropriate dilution (Table 1) and phalloidin–rhodamine (1:200, Thermo Fisher Scientific, France) or Lectin-FITC (1:200, L32478, Invitrogen™ Thermo Fisher Scientific, France) in a buffer solution (PBS 0.1M, 5% normal donkey serum, 0.01% Triton X-100) during 5 days at 4°C under gentle agitation. After 6 X 30 min consecutive washings with 0.1M PBS / 0.01% Triton X-100, tissues were incubated for 3 h at RT with the adequate secondary antibodies diluted at 1:1000. After 4 X 30min successive washings, tissues were flat-mounted using Dako Omnis Fluorescence Mounting Medium (Agilent, Les Ulis, France). Similar protocol was applied for immunostaining on transversal cryosections, with overnight (4 °C) incubation for the primary antibody and 1 h (at RT) for secondary antibody incubation. Images were acquired using a fluorescence microscope (model Olympus BX51, Olympus, Rungis, France) or a confocal microscope (model Zeiss LSM710, Zeiss, Rueil Malmaison, France).

**Table 1:** List of antibodies

Antigen	Supplier	Reference	Host specie	Dilution	Targeted structure
TUBB3	Biolegend, San Diego, United States	801202	Mouse	1:500	Neuronal soma, axon and dendrite
IBA1	Fujifilm Wako, Neuss, Germany	019-19741	Rabbit	1:500	Macrophages
NPY	Cell signalling, Danvers, United States	CST11976	Rabbit	1:500	Sympathetic fibres
αSMA	Abcam, Cambridge, England	ab5694	Rabbit	1:100	Vascular walls, arteries and veins
Synaptophysin		ab8049	Mouse	1:500	Synapses
VIP		ab22736	Rabbit	1:500	Parasympathetic fibres
CGRP		ab81887	Mouse	1:200	Sensory nervous fibres
PGP9.5		ab108986	Rabbit	1:500	Neuronal soma, axon and dendrite
NF200	Sigma-Aldrich Darmstadt, Germany	N-4142	Rabbit	1:100	Neuronal soma, axon and dendrite
ChAT		AB-144P	Goat	1:200	Parasympathetic fibres
Collagen IV	Novusbio, Centennial, United States	NB120-6586	Rabbit	1:100	Choroidal vessels

### Tissue preparation and transcriptomics analysis

Five-month-old male P1.hMR mice (n = 3, 6 eyes) and their age- and sex-matched wild-type (WT) littermates (n = 2, 4 eyes) were euthanized, eyes were removed and dissected on ice to isolate the sclera–choroid–RPE complexes, which were immediately frozen in liquid nitrogen (2 eyes from the same animal were pooled). RNA extraction was performed within a few days using Qiagen RNeasy KIT (Cat. 74004, Qiagen©, Hombrechitikon, Switzerland), and 500 ng of extracted RNA was sent for RNA sequencing at the iGenSeq transcriptomic platform of the Brain and Spine Institute (ICM, Paris, France). Quality of raw data was evaluated with FastQC. Poor-quality sequences and adapters were trimmed or removed with fastp, using default parameters, to retain only good-quality paired reads. Illumina DRAGEN bio-IT Plateform (v3.8.4) was used for mapping on mm10 reference genome and quantification with genecode vM25 annotation gtf file. Library orientation, library composition and coverage along transcripts were checked with Picard tools. The transcriptomic data were analysed on the online platform of the Paris Brain institute (quby.icm-institute.org). First, differential expression analysis using edgeR method was assessed to determine the differentially expressed genes (adjusted P-value (pFDR), FDR threshold set at 0.05, log2 fold-change threshold set at 0.5). Then, an enrichment analysis was performed (by over-representation analysis) in different gene-set collections (MSigDB database) including Hallmark gene sets (H), Reactome gene sets (Reactome subset of Canonical pathways) and Gene Ontology gene sets (C5:GO).

### Mouse tissues preparation for semi-thin and transmission electron microscopy (TEM)

Five-month-old male and female P1.hMR mice (*n* = 3) and their WT littermate (*n* = 3) were euthanized, and eyes were fixed with 2.5% glutaraldehyde for histological and TEM studies. Briefly, eyes were rinsed in cacodylate buffer for 2 h before being post-fixed with osmium tetroxide (OsO4 2% in cacodylate buffer). Then, samples were dehydrated in a graded alcohol series (70%, 90% and 100%) for 15 min each, before being embedded using LX112 embedding kit (Ladd Research Industries, Williston, United States). The polymerization occurred at 60 °C for 48 h. Blocks were finally cut using an ultramicrotome, either for semi-thin section (1 µm thickness and stained with toluidine blue) or ultrathin section (80 nm). Ultrathin sections were then contrasted and observed with a TEM.

### Serial-block-face Scanning electron microscopy (SBF-SEM)

SEM-SBF study was conducted at the Jacques Monod institute ImagoSeine platform (Université Paris Cité, CNRS). Mouse eyes were fixed with a mix of 1% glutaraldehyde and 3% paraformaldehyde (*n* = 6, 3 P1.hMR and 3 WT mice). Samples were washed 3 times with 1X PBS and post-fixed for 1 h in a reduced osmium solution containing 1% osmium tetroxide, 1.5% potassium ferrocyanide in PBS1X buffer, followed by incubation with a 1% thio-carbo-hydrazide (TCH) solution in water for 20 min at RT. Subsequently, samples were fixed with 2% OsO4 in water for 30 min at RT, followed by 1% aqueous uranyl acetate at 4 °C overnight. The samples were then subjected to bloc Walton’s lead aspartate staining and placed in a 60 °C oven for 30 min. Samples were then dehydrated in graded concentrations of ethanol for 10 min each. The samples were infiltrated with 50% Agar low viscosity resin (Agar Scientific Ltd) overnight or 2 h and 100% Agar low viscosity resin overnight. The resin was then changed with 100% Agar low viscosity for 1 h, two times and the samples further incubated during 1 h prior to polymerization for 18 h at 60 °C. The polymerized samples were mounted onto special aluminium pins for SBF imaging (FEI Microtome 8 mm SEM Stub, Agar Scientific), with two-part conduction silver epoxy kit (EMS, 190,215). Mounted samples were trimmed and inserted into a TeneoVS SEM (Thermo Fisher Scientific). Acquisitions were performed with a beam energy of 2 kV, a current of 200 pA, in LowVac mode at 40 Pa, a dwell time of 1 µs per pixel, a pixel size of 10 nm and sections of 100 nm. The scanning was focussed on large choroidal nerves and nearby choroidal fibres. Data alignment and segmentation were performed using Amira software (Thermo Scientific™).

### Clinical multimodal imaging analysis

Clinical images from one patient diagnosed with complex CSCR and widespread pigment epitheliopathy were collected. The study adhered to the tenets of Declaration of Helsinki and the French legislation. Indocyanine green angiography (ICG-A) and fluorescein angiography (FA) were performed with Spectralis (Heidelberg Engineering, Heidelberg, Germany). Spectral-domain optical coherence tomography (SD-OCT) with enhanced depth imaging (EDI) was carried out on the same machine, using the built-in calliper for measurements. Infrared en-face images (IR) were obtained simultaneously with the SD-OCT as part of the routine predefined acquisition module.

## Results

### Anatomical and neurochemical characterisation of rat choroidal nervous system (ChNS)

**Fig. 1:**
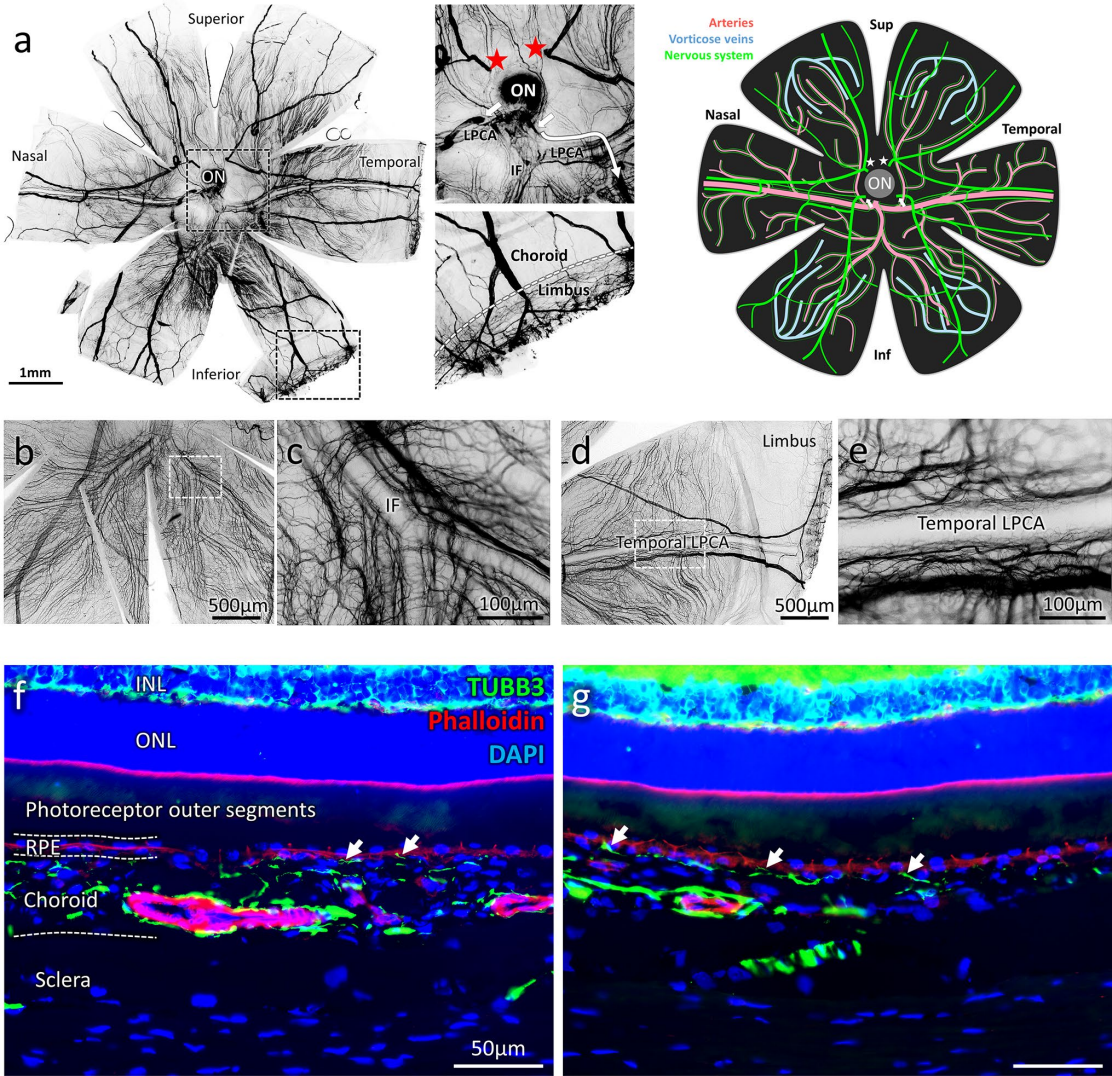
Visualisation and general organization of the choroidal nervous system in rats. **a** Example of a rat choroid stained with TUBB3 after negative filtering using Image J, revealing the general organisation of the choroidal nervous system (ChNS). The two pictures respectively represent a zoomed view of the optic nerve (ON) zone and the transition zone between the choroid and the limbus. The arrows and stars indicate large nerves entering the choroid. Two large nerves are entering in nasal and superior parts of the choroid (stars) whilst two others seem to enter in the nasal and temporal inferior part (arrows). The inferior temporal nerve is out of focus on the long arrow trajectory, remerging just under the LPCA. The zoomed view of the peripheral choroid is showing that the large nerves are responsible for the limbus/corneal innervation. These large fibres are probably corresponding to the human short and long ciliary nerves. Schematic representation of the ChNS (in green) organisation around the vascular system (in pink and blue). Pictures **b–e** represent the organisation of the ChNS surrounding the IF and the temporal LPCA. **b, c** Architecture of the IF innervation, the fibres originating in the superior IF bifurcates progressively following the arterial bifurcations. The large nerve (coming from the nasal inferior zone around the optic nerve) is passing under the IF, making collaterals. **d, e** Architecture of the temporal LPCA innervation, the fibres are surrounding the LPCA and bifurcate progressively at arterial-arteriole bifurcations. Large nerves follow the trajectory of the LPCA toward the peripheral choroid and innervate the limbus/cornea. **f, g** TUBB3 immunostaining (in green) and phalloidin-rhodamine staining (in red) reveal the ChNS organisation on rat eyes transversal sections. The nerve fibres are strongly organised around the arteries (strongly stained with the phalloidin) and numerous fibres can be observed in the choriocapillaris, juxtaposed to the RPE (white arrows). ON: Optic nerve; LPCA: Long posterior ciliary arteries; IF: Inferior branch

In albinos rats, tubulin β3 (TUBB3) staining on flat-mounted eye fundus allows the visualisation of the entire nerve network within the choroid (Fig. 1). Whilst the general ChNS organisation displays similarities between individuals and a typical pattern, inter-individual variations are noticeable in the distribution of nerve ramifications. Animals from the same age and sex might display differences in the trajectories, diameters and even in presence/absence of large nerves. Nonetheless, a general organisation shared by most animals allows to draw a schematic pattern of the rat ChNS anatomy (Fig. 1a scheme).

The largest nerves are entering the choroid nearby the optic nerve in the superior and in the inferior part of the choroid (Fig. 1a), corresponding in humans to short and long ciliary nerves. Most of the largest fibres follow a radial organisation, starting from the optic nerve zone to the periphery. Two large nerves emerging just above the optic nerve (stars, Fig. 1a) and generally bifurcate either towards the superior choroid or to the long posterior ciliary arteries (LPCA). Nerves nearby the LPCA form “bridge”-like structures, passing above the LPCA. Then, these large nerves either continue all along the LPCA or bifurcate and go straight respectively to the nasal and temporal inferior part of the choroid. Two others large nerves emerge from a common origin nearby the inferior nasal and temporal zone of the optic nerve respectively (arrows, Fig. 1a). These nerves pass above the LPCA and bifurcate to either follow the LPCA trajectory, joining the fibres which come from the superior choroid, or go through the inferior choroid making ramifications at the periphery. This part of the ChNS innervates the inferior branch (IF) as well (Fig. 1a–d). If these large choroidal nerves follow a radial organisation, they are also connected to each other through large “perpendicular” bifurcations (Fig. 1a) in the manner of a “spider web”, notably in the inferior part of the choroid. Thus, large radial choroidal nerves are forming an important network including large primary routes and thinner secondary connexions. This network seems to be complementary to the choroidal innervation which arises from the ciliary artery.

**Fig. 2:**
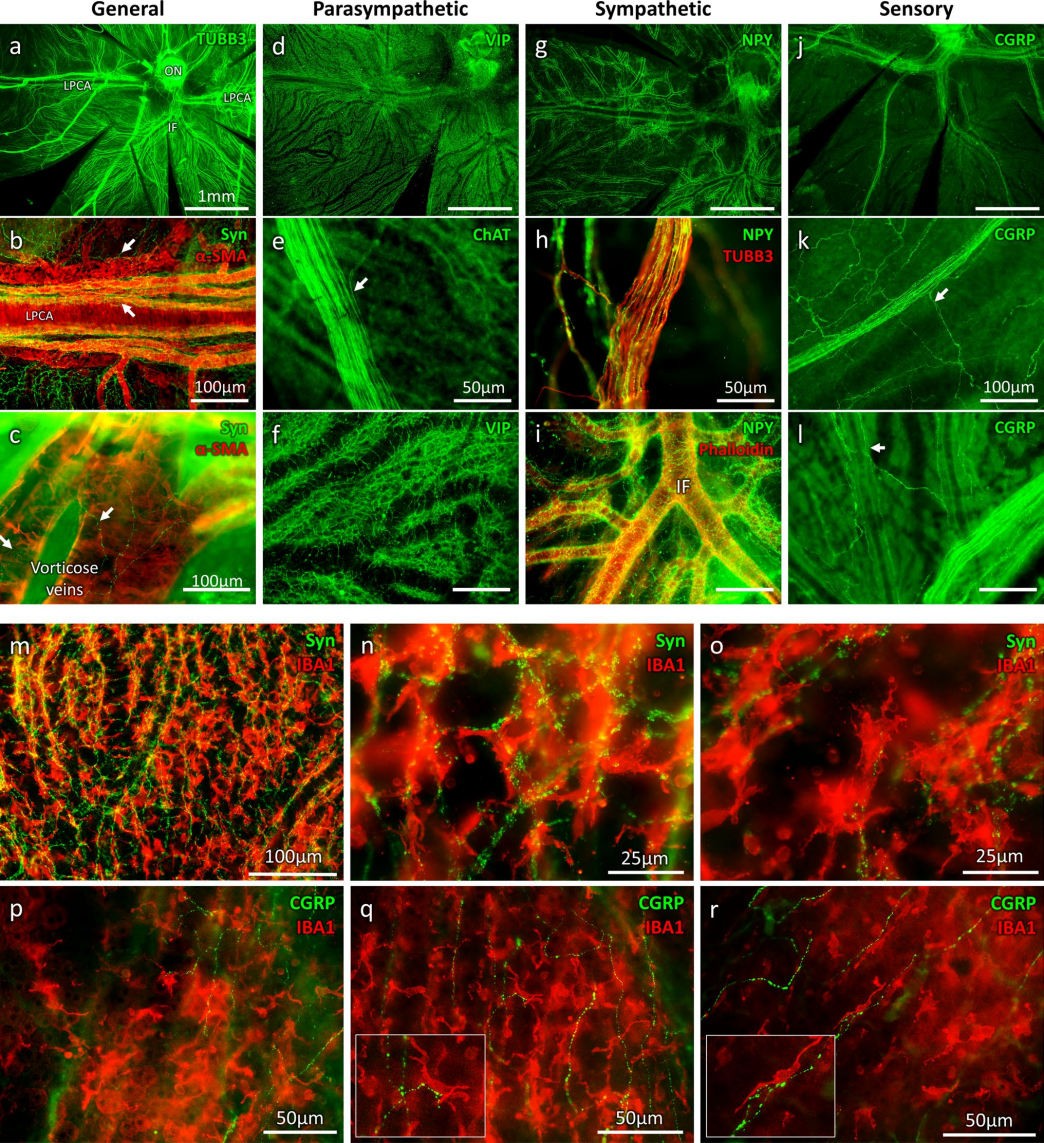
ChNS neurochemical profiles, vascular targets, and contact with resident macrophages. TUBB3/Synaptophysin (neuronal and synaptic marker) VIP/ChAT (parasympathetic) NPY (sympathetic), CGRP (sensory) phalloidin/α-SMA (vascular markers) were used to visualise the neurochemical profile and targets of the choroidal innervation. **a–c** General organisation of the ChNS showing important vascular targeting, both arterial and venous structure are innervated. **d–f** Parasympathetic (VIP/ChAT) component is the major component of the ChNS. Parasympathetic fibres are found in the ciliary nerves (**e**), forming sometimes bifurcations (arrow) which innervate the choroid. VIP-positive fibres are targeting the vessels and are also highly represented in the intervascular space. **g–i** Sympathetic nervous system is the second major component of the ChNS. It is present in the ciliary nerves (**h**) and is almost exclusively organised around the choroidal vessels. **j–l** Sensory nervous system is the less represented component of the ChNS. It is highly present in the ciliary nerves, which form multiple bifurcations innervating the choroid. The CGRP-positive fibres are targeting the choroidal vessels but are also found in the intervascular space. **m–o** IBA1 and Synaptophysin immunostaining reveals the anatomical proximity between the choroidal macrophages and the choroidal nervous system. Numerous immune cells are organised around the vessels, in different layers, and close to the surrounding synapses. Numerous synaptophysin-positive zones are found in contact with IBA1-positive cells. **p–r** CGRP-positive fibres are found close to immune cells, sometimes displaying apparent contacts, which suggest an interaction between the immune system and the sensory nervous system within the choroid

Whilst ciliary nerves make collaterals to innervate nearby choroidal structures, their diameters remain quite constant along their trajectory up to the limbus/cornea. Hence, the choroid is largely innervated by fibres entering the choroid with the ciliary artery nearby the optic nerve and partially by fibres from ciliary nerves along their trajectory through the choroid (Fig. 2). Innervation is dense around the arterial network (the LPCA and the IF being densely innervated), bifurcating gradually at the arteries–arterioles transitions (Fig. 1b–e) with gradual density decrease toward the periphery. Interestingly, significant innervation is also found around the veins (Figs. 1, 2). On choroid/retina sections (Fig. 1f, g), TUBB3 used to identify the nerves, shows fibres located also around the choriocapillaris and up to the RPE cells, suggesting that the choriocapillaris and the RPE receive ChNS inputs.

Immunostaining on flat-mounted choroid identifies the neurochemical nature of the sympathetic, parasympathetic, and sensory fibres (Fig. 2). VIP/ChAT-positive fibres, NPY-positive fibres and CGRP-positive fibres are all found within the large nerves/ciliary nerves (Fig. 2e–k). Furthermore, the three markers are robustly expressed all along the ciliary nerves until the peripheral choroid, indicating a strong role of the autonomous and sensory nervous systems within the limbus/cornea. Numerous ciliary nerves bifurcations entering the choroid are CGRP-positive. Outside the ciliary nerves, the parasympathetic nervous system is the most represented component of the choroidal innervation (Fig. 2d–f), consistent with a previous functional study on the need for an important choroidal/outer retinal oxygenation [62]. VIP-positive fibres are organised around the arterial system, including the LPCA and around the venous system and in the intervascular space. The sympathetic nervous system (Fig. 2g–i) is present in the ciliary nerves and is almost exclusively organised around the choroidal vessels (artery and veins), with very few NPY-positive fibres found in the intervascular space. Finally, the sensory nervous system is the less represented part of the ChNS (Fig. 2j–l), but is dense within the ciliary nerves, forming multiple bifurcations innervating the choroid. CGRP-positive fibres are distributed around the choroidal vessels and in the intervascular space.

Around choroidal vessels, together with the dense network of IBA1-positive macrophages, synaptophysin staining shows the dense and well-organised network of synapses that seems to coordinate the immune cross-talk between nerves, vessels and macrophages (Fig. 2m–o). More specifically, CGRP-positive vesicle were aligned along macrophages, suggesting that CGRP modulates macrophage activity (Fig. 2p–r). These observations highlight a neurogenic control of the choroidal innate immune system, including through sensory regulation.

**Fig. 3:**
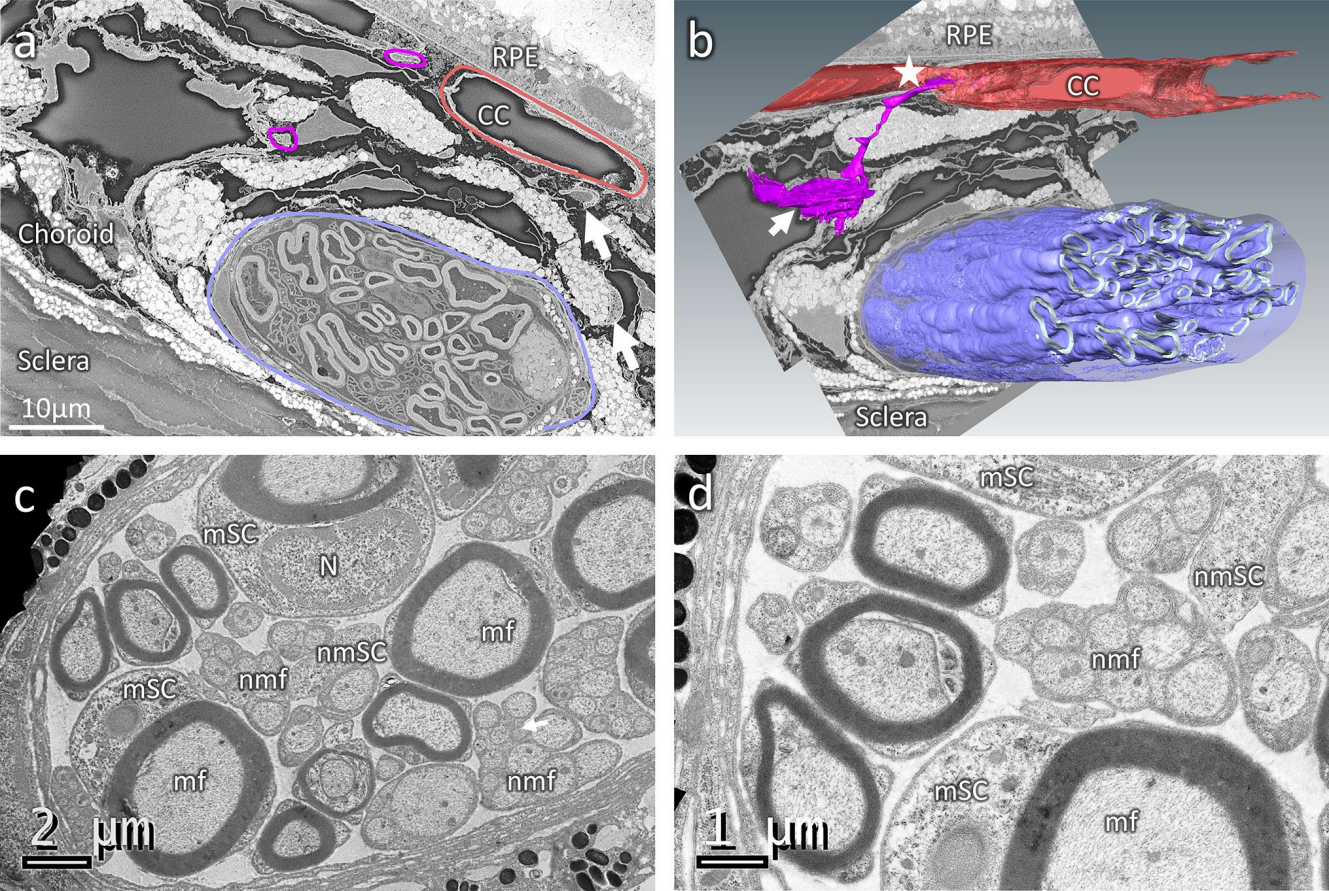
Ultrastructure of mouse choroidal innervation by SBF-SEM and TEM. **a** A single section of the mouse choroid from a volume acquired by SBF-SEM, showing a large choroidal nerve (purple), choroidal nerve fibres (magenta) and the choriocapillaris (CC, in red). Other small fibres are visible (arrows) as well as pigments from melanocytes (white) and large vessels (dark). **b** 3D reconstruction of the SBF-SEM volume presented in A, with the myelin of the large nerve (purple), the choroidal nerve fibre (magenta) and the choriocapillaris (red) segmented. The reconstruction reveals the ultrastructural organisation of the choroidal innervation, with a clear innervation of large vessels (arrow) and CC/RPE (star). **c, d** Transmission electron microscopy (TEM) analysis of a large choroidal nerve after osmium contrasting. The large nerve is composed by large, myelinated fibres (mf) nearby myelinating Schwann cells (mSC) and group of non-myelinated fibres (nmf) where non-myelinating Schwann cells (nmSC) are visible. N: myelinated Schwann cell nucleus

Ultrastructural analysis of the rodent choroidal innervation was performed using transmission electron microscopy (TEM) and Serial Block Face—Scanning Electron Microscopy (SBF-SEM) (Fig. 3). It shows that large choroidal nerves contain both large, myelinated fibres and numerous groups of non-myelinated fibres (Fig. 3a, c, d), together with adjacent myelinating Schwann cells (mSC) and non-myelinating Schwann cells (nmSC). Outside of ciliary nerves, the non-myelinated fibres that fill the intervascular space extend anteriorly to innervate the choriocapillaris (Fig. 3a, b) as shown by SBF-SEM, which allows the tracking and the 3D reconstruction of these fibres (Fig. 3b—Supplementary file 1), confirming our immunostaining findings on sections (Fig. 1e, f).

### Anatomical and neurochemical characterisation of the human choroidal nervous system

**Fig. 4:**
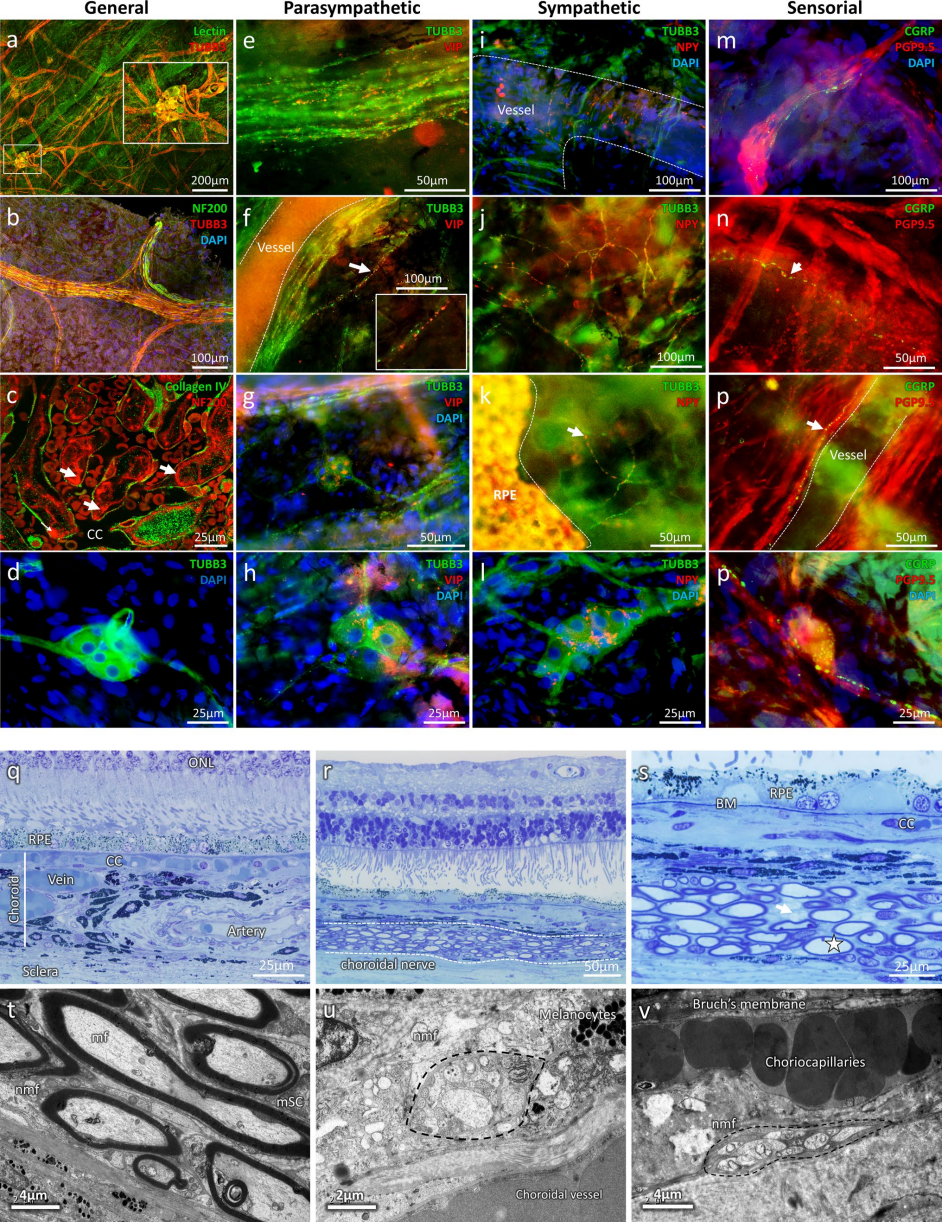
Immunohistochemical and ultrastructural analysis of the human choroidal nervous system. **a** General organisation of the human choroidal nervous system (TUBB3) and its proximity with the choroidal vessels (Lectin), iChNS are clearly noticeable. **b** large choroidal nerve corresponding to human ciliary nerve. **c** NF200 is found in the inter-capillary space (white arrows), showing innervation of the choriocapillaris (CC) (Collagen IV). (**d**) TUBB3 staining allows the visualisation of IChNS and their neurites. **e–h** Parasympathetic fibres and VIP-positive IChNS (**g-h**) are present in the human choroid, both in large nerve (**e**) and around choroidal vessels (**f**). **i–l** Sympathetic fibres are numerous in the human choroid, both around large vessels (**I**) but also at the level of the CC, just under the RPE (**k**). Some IChNS were also found positive for NPY (**l**). **m–p** Sensory fibres are rare and are noticeable in large nerve fibres (**m**) and around choroidal vessels (**n–o**). Few IChNS carry CGRP-vesicles, indicating sensory properties (**p**). **q–s** Transversal Semithin sections of healthy aged human eye. Large choroidal nerves are easily noticeable (**r, s**) with myelinated (**s,** white star) and unmyelinated fibres (**s**, white arrow). **t, u** TEM observation of transversal section of human choroid. **t** Large choroidal nerve containing myelinated fibres (mf) and their myelinating Schwann cell (mSC) as well as non-myelinated fibres (nmf). **u** Non-myelinated choroidal fibres nearby a choroidal vessel. **v** Non-myelinated choroidal fibres contacting the choriocapillaris

**Fig. 5:**
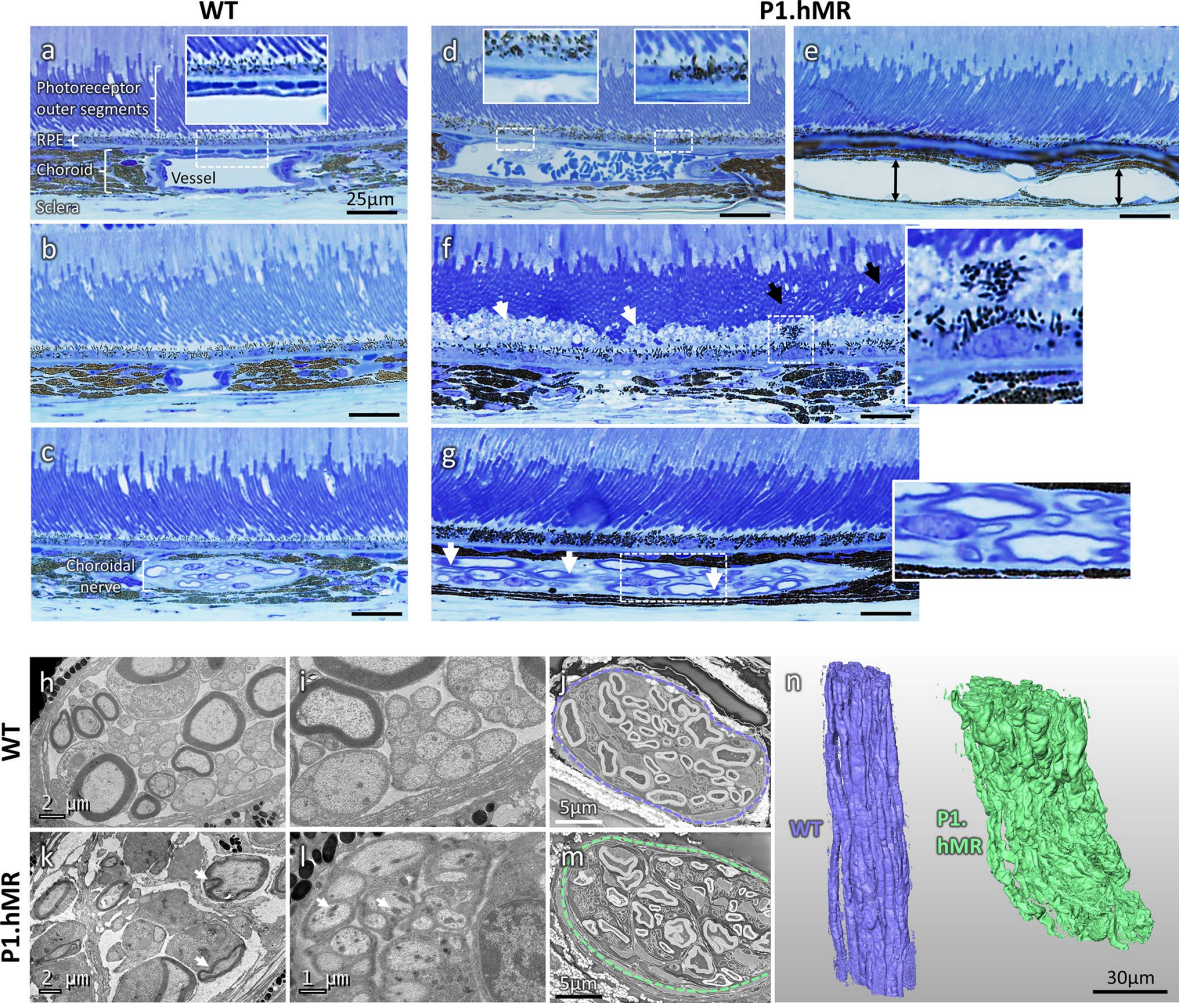
Semithin and ultrastructural analysis of P1.hMR and WT littermate mice choroid and choroidal nerves. **a–c** Semithin transversal eye section of WT littermate mice. The photoreceptor outer segments are aligned and the choriocapillaris are clearly noticeable between large choroidal artery and RPE (**a**). RPE pigments are concentrated and well spread toward the apical pole of the cells (**a** inset and** b**). Choroidal nerve displays healthy structure with normal myelin organisation (**c**). (**d–g**) Semithin transversal eye section of transgenic P1.hMR mice where several features of pachychoroid are observed. Dilated vessels in direct contact with Bruch membrane and effacement of the overlying choriocapillaris (“pachyvessel”) (**d,** left inset) associated with irregular pigment distribution in the RPE (**d,** right inset). Dilated veins (**e,** black double arrows), with focal area of elongated undigested photoreceptor outer segments (**f,** black arrows) and subretinal deposits associated with abnormal RPE/photoreceptor outer segment interface (**f**, white arrows). These are accompanied by irregular pigment distribution in the RPE and subretinal migration of RPE cells (**f**, inset). These features resemble pachychoroid epitheliopathy described in humans. In P1.hMR, enlarged nerves with irregular myelin shedding (white arrows) and disorganisation were observed (**g**). These neural abnormalities were more obvious in electron microscopy observations. TEM of WT littermate (**h, i**) and transgenic P1.hMR mice (**k, l**) choroidal nerves**.** Transgenic mice display signs of neuropathy, including myelin abnormalities (**k**, white arrows), increased number of large mitochondria (**l**, white arrows) and loss of organisation and vacuolization of the nerve. SBF-SEM sections of a choroidal nerve in a WT littermate (**j**, purple area) or in a transgenic mouse (**m**, green area), and the corresponding 3D segmentation of the myelin (**n**). Myelin abnormalities in transgenic animals are clearly apparent, including large variation of the G-ratio

Human peripheral and macular choroid have been obtained by dissection, after gently removing the neuroretina, the RPE and the sclera (Supplementary Fig. 2). TUBB3, PGP9.5 and NF200 marker have been used to label the nerves whilst Lectin & collagen IV were used to stain the choroidal vasculature (Fig. 4—Supplementary Fig. 2). Aside from the large, long and short ciliary nerves, (Fig. 4b) a very dense and ramified nervous system innervates the choroidal vasculature with visible intrinsic choroidal neurons (IChNS) (Fig. 5a–d). Unexpectedly, numerous nerve fibres (NF200-positive) innervate the choriocapillaris (white arrows, Fig. 4c). Neurochemical characterisation shows that choroidal vessels receive sensory (CGRP-positive fibres, Fig. 4m–o), autonomic sympathetic (NPY-positive fibres, Fig. 4i, j) and parasympathetic (VIP-positive fibres, Fig. 4e, f) inputs, consistent with what we observed in rats. Interestingly, NPY-positive fibres are found to connect with the choriocapillaris, just under the RPE (Fig. 4k). In addition to the sensory and autonomic nervous network described before, numerous IChNS are in the macular and the peripheral zone. These neurons with round-shaped nucleus can be found alone or organised in plexi of two or more cells, displaying bipolar or multipolar organisation. They are also connected between them, forming their own network within the choroid, and targeting the choroidal vasculature (Fig. 4a and inset). Neurochemical characterisation revealed IChNS containing VIP-vesicles (Fig. 4g, h), and CGRP-positive vesicles (Fig. 4p), in accordance with the literature. However, NPY-positive vesicles (Fig. 4l) were also clearly found within the soma, revealing potential sympathetic properties.

Similar to our observations in rodents, ultrastructural analysis by TEM shows large nerves formed by myelinated and unmyelinated fibres, together with myelinated and non-myelinated Schwann cells (Fig. 4t). Numerous non-myelinated fibres are in close contact with all vessel types including arteries, veins, and choriocapillaris (Fig. 4u, v).

### hMR overexpression is associated with pachychoroid-like phenotype and with choroidal neuropathy

In transgenic mice overexpressing hMR, several features of chorioretinopathy are observed in semithin sections, in different areas as compared to WT littermates (Fig. 5). Vascular abnormalities include dilated veins with focal area in direct contact with the Bruch membrane, leading to an effacement of the choriocapillaris (Fig. 5d), similar to pachyvessels described in humans in spectral domain optical coherence tomography. Abnormal melanosome organisation is found in RPE cells with irregular distribution and area of aggregation (Fig. 5d–f). The large choroidal nerves show signs of neuropathy, such as fibres enlargement and myelin disorganisation (Fig. 5g).

In the retina, the most frequent abnormalities in transgenic animals were focal area of elongated undigested photoreceptor outer segments and subretinal deposits (Fig. 5f). These subretinal deposits were found associated with irregular pigment distribution in the RPE and subretinal migration of RPE cells.

In order to mimic the clinical situation in humans that favours the onset of CRSC, we exposed WT and P1.hMR transgenic mice to a long-acting glucocorticoid, triamcinolone acetonide (TA), injected sub-conjunctivally. At 8 days, no morphological abnormalities of the retina or choroid were observed in WT mice. In contrast, injection of TA into P1.hMR mice increased the signs of choroidopathy. Choroidal vasodilation was associated with areas of RPE cell migration, proliferation and pigment dispersion (Supplementary Fig. 3b, inset) and to RPE folds (Supplementary Fig. 3c star, e arrows, f, g arrow). Major elongation of undigested photoreceptor outer segments (Supplementary Fig. 3c-f) was associated with focal area of detachments, which could result from the section process in fragile area or to real serous detachments (Supplementary Fig. 3c-f).

TEM observations in transgenic animals confirm clear signs of neuropathy including myelin and fibres disorganisation as well as vacuolization and mitochondrial abnormalities. Myelin abnormalities include local demyelination and important variations in the ratio between fibres thickness and myelin sheet thickness, which are not present in littermates (Fig. 5h, i, k, l). Large fibres also lost their circular shapes and are often found flattened (Fig. 5k), with vacuolization and unmyelinated disorganised fibres. Finally, TEM analysis of transgenic mice also showed an increased number of large and osmium-labelled mitochondria inside both myelinated and unmyelinated fibres (Fig. 5l), constituting a characteristic sign of neuropathy. SBF-SEM acquisitions were used to visualise in 3D the myelin organisation in large choroidal nerves of transgenic and littermate animals (Fig. 5j, m, n). These reconstructions were consistent with the previous observations in TEM, confirming the myelin disorganizations and irregularity along the nerve in P1.hMR mice (Fig. 5n). Typical ultrastructural signs of choroidal neuropathy result from mineralocorticoid receptor pathway overactivation in transgenic mice.

**Fig. 6:**
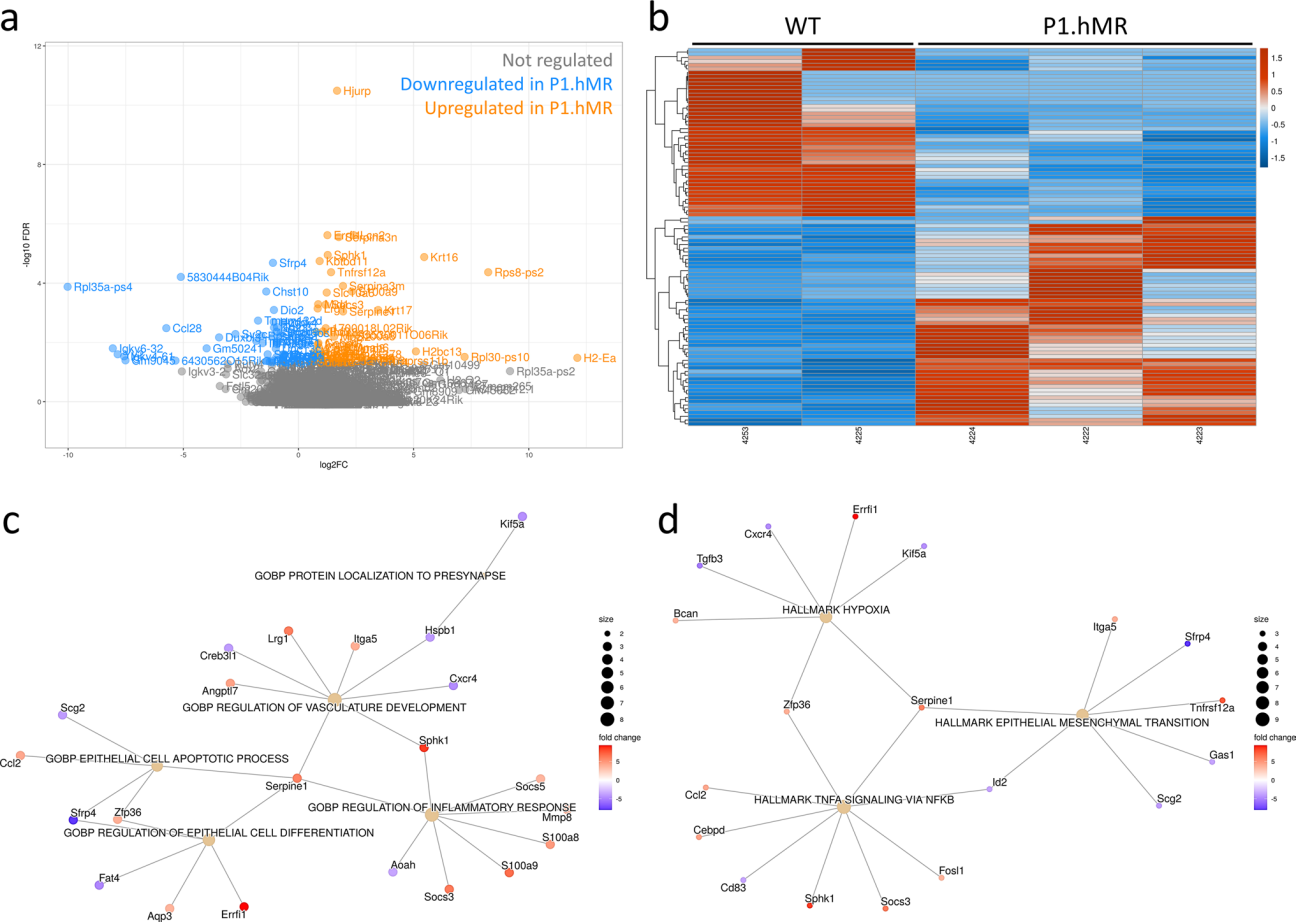
Differential transcriptomic analysis and gene concept network for differentially expressed genes in P1.hMR mice versus WT mice. Volcano plot (**a**) and heat map (**b**) display 45 significantly downregulated and 56 upregulated genes in P1.hMR mice compared to their age/sex-matched WT littermates. **c** Significantly regulated pathways in Gene Ontology gene sets (C5:GO) enrichment analysis. **d** Significantly regulated pathways in hallmark gene sets (H)

To explore the underlying mechanisms of MR-induced choroidopathy, bulk RNA transcriptome was performed to compare the sclera-choroid-RPE genes expression between P1.hMR mice and their littermates (Fig. 6). After differential analysis using edgeR method, 101 genes were found differentially expressed in transgenic animals (raw data available on Gene Expression Omnibus [29] with complete gene list in Supplementary file 2), with 45 downregulated and 56 upregulated. The enrichment analysis in GO gene sets related to biological process revealed that pathways related to protein localization to presynapse (*pFDR* = 0.04340), vasculature development (*pFDR* = 0.00797), regulation of epithelial cell differentiation (*pFDR* = 0.00560), epithelial cell apoptotic process (*pFDR* = 0.01070) as well as regulation of inflammatory response (*pFDR* = 0.01020) were significantly regulated in P1.hMR animals compared to their WT littermates (Fig. 6c). In addition, analysis of Hallmark gene sets also showed three significantly regulated biological processes in transgenic animals, including hypoxia (*pFDR* = 0.02578), epithelial mesenchymal transition (*pFDR* = 0.002578) and TNFα signalling via NF-κB (*pFDR* = 0.00188) (Fig. 6d).

### Choroidal phenotype in patients with pachychoroid-associated central serous chorioretinopathy (CSCR)

**Fig. 7:**
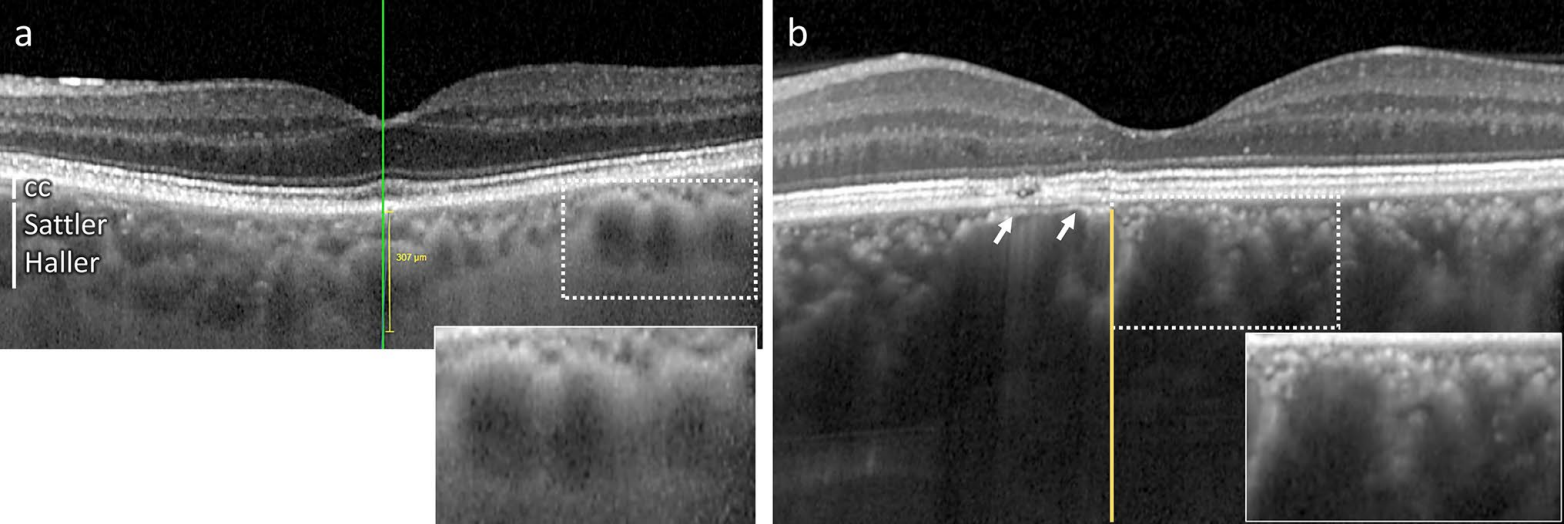
Foveal SD-OCT in healthy and CSCR conditions. **a** Foveal SD-OCT of a healthy retina, showing a thickened choroid with dilated choroidal vessels. In the enlarged quadrant, vessels are visualised as round hyporeflective structures with a homogenous hyperreflective border, more pronounced towards the RPE. The choriocapillaris remains visible despite choroidal enlargement. **b** In this chronic resolving CSCR, pachychoroid is observed, as reported on SD-OCT of the left eye. An enlarged choroidal vessel in the perifoveal zone is visualised underneath the RPE and prevents correct visualisation of the choriocapillaris (white arrows), associated with discrete RPE elevation and outer retina displacement. In the enlarged quadrant, several hyperreflective dots are observed, both surrounding large choroidal vessels and within the choriocapillary space, with a granular irregular appearance

**Fig. 8:**
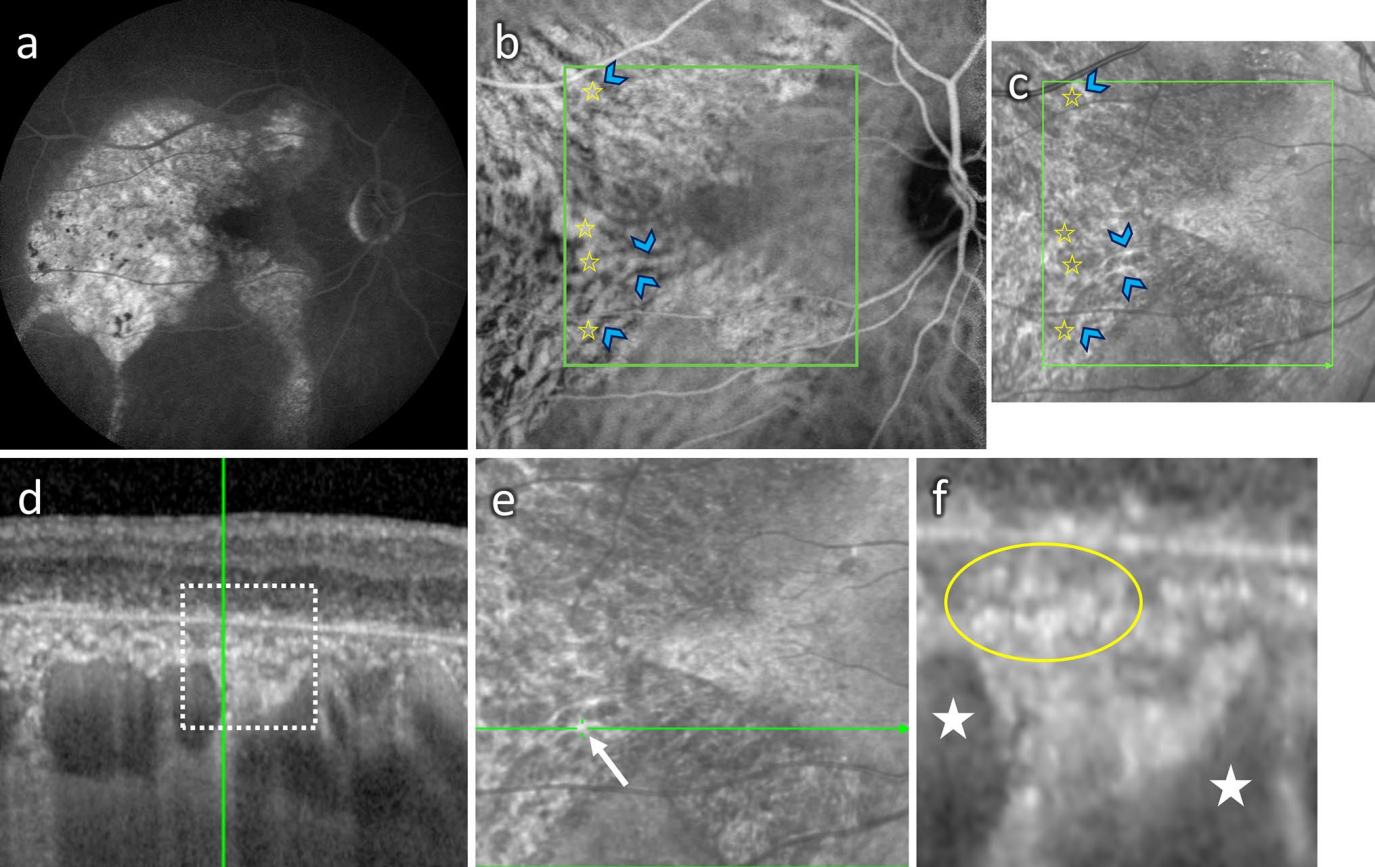
Clinical features on multimodal imaging of a 55-year-old man with chronic CSCR and widespread pigment epitheliopathy. The RPE atrophy is highlighted by the extensive window defect shown on early-phase fluorescein angiography (**a**), which allowed a more detailed visualisation of choroidal vasculature in the early-phase IGC cliché at 1 min (**b**), showing the dye in the intravascular space. On infrared en-face imaging (**c**), choroidal vessels were visualised as dark areas (yellow star). Adjacent to choroidal vasculature dark reflectance, white linear structures with increased IR reflectance were observed (blue arrowhead). These were hypofluorescent on ICG (**b,** corresponding yellow stars and arrowheads). **d** SD-OCT EDI scan showing photoreceptor disorganisation and RPE loss, pachychoroid and enlarged choroidal vessels. The green vertical line shows that the B-scan is placed over the white linear structure in the en-face IR image (**e**, white arrow). The corresponding location on SD-OCT (**f**, enlarged image) shows a hyperreflective area (yellow circle) very close to enlarged choroidal vessels (white star), underneath the choriocapillaris

In a normal choroid, large vessel diameters are highly variable. Despite being large and protruding anteriorly, the overlying choriocapillaris remains visible as a thin hyporeflective layer in SD-OCT (Fig. 7a). In eyes with pachychoroid-associated diseases, vascular dilation can be extremely important, leading to choriocapillary compression, and extending up to Bruch’s membrane, in areas where the choriocapillaris cannot be distinguished and the overlying RPE forms bumps, displacing the outer retinal bands (Fig. 7b**,** arrows). On top of this typical sign, the vessels are surrounded by irregularly shaped hyperreflective structures with blisters, distinct from hyperreflective dots as they do not vary with time or with activity of the disease. Such irregular perivascular hyperreflectivity is very characteristic of this pathology, as previously described [24], and is present in patients whose RPE cells and outer retina are still maintained, as shown in Fig. 7B of this middle-aged men who presented with resolving CSCR in his left eye. In more advanced and complex CSCR [21], atrophy of the RPE and the outer retina allows a more direct visualisation on the choroid, with increased light passing through. An example of such patient is shown in Fig. 8, where RPE atrophy is highlighted by the extensive window defect shown in the early-phase fluorescein angiography (Fig. 8a), the highly visible vessels on early-phase indocyanine green (ICG) angiography (Fig. 8c**)** and on B-scan SD-OCT with photoreceptor disorganisation and RPE loss (Fig. 8e). On the infrared image, large choroidal vessels appear dark, surrounded by a white filamentous network particularly dense around vessels (Fig. 8b). Superposition on infrared image with ICG angiography clearly shows that the hyperreflective network does not correspond to vessels but surround and / or cover them (Fig. 8b). On SD-OCT B-scan (Fig. 8e, f), the exact location of one hyperreflective filamentous structure corresponds to a perivascular elongated and individualised band. Comparison with the histological organisation of the ChNS highly suggests that those hyperreflective structures, visible on infrared imaging and SD-OCT correspond to pathological nerves, similar to the irregular and dilated nerves imaged in P1.hMR using electron microscopy. The size of the nerves observed in humans as compared to choroidal vessels is compatible with the size of images observed on clinical images.

## Discussion

### On the importance of the choroidal nervous system in all choroidal functions

In pachychoroid spectrum diseases, choroidal clinical markers and therapeutic targets are focussed on the vasculature (pachyvessels [18], venous overload [66], choroidal neovascularization [59], vascular leakage [38] and inflammation (hyperreflective dots, RPE leak, oedema), with little to no attention to the choroidal neural system, although choroidal vasculature in under neural control [62].

In this paper, we firstly identified that in addition to innervation of arteries that is well known, choroidal veins (including vorticose veins) and choriocapillaris (CC) are also innervated both in humans and rodents. This suggests that ChNS could play the role of a “coordinator”, which not only actively regulates the inflow (arteries) and the outflow (vorticose veins), but also the flow in the CC. This could have important implications in pathology, as “venous overload” and CC occlusion would thus be the consequence of an active neuronal deregulation.

Our neurochemical analysis confirms previous observations on the nature of ganglions which project to the choroid. Autonomous (sympathetic and parasympathetic) nervous system is mainly organised around the choroidal vasculature with a clear predominance of the parasympathetic component, consistent with Reiner’s team view on the importance of vasodilatation to avoid under-perfusion of the choroid. On the other hand, the sensory nervous system displays a distinct organisation, with fibres around choroidal vessels and others covering non-vascularized area. As mechanical and physical stresses (temperature and PH variation) are potential regulators of choroidal blood flow, the CGRP fibres could strategically sensitise specific choroidal zones, to efficiently detect and rapidly respond to such stimuli. Interestingly, numerous CGRP-fibres are provided from large choroidal nerve bifurcations. Hence, sensory signal would be retrogradely transmitted and could affect large choroidal zones by travelling transversely through secondary routes.

In addition to vessel innervation, our finding strongly suggests a CGRP-mediated neuro-immune cross-talk in the choroid, because CGRP-vesicles are juxtaposed to macrophages as it has been shown in other tissues [22]. Such cross-talk could have intervened in the inhibitory effect of CGRP receptor antibodies in choroidal neovascularization [50]. In addition, the numerous non-CGRP-positive fibres connecting with macrophages implies that the neural crosstalk also involves autonomous regulation, and that the choroid could be the site of neurogenic inflammation.

Interestingly, besides the parasympathetic and sensory IChNS, described in humans [27, 32, 61, 73], we observed NPY-positive IChNS. This indicates either the existence of some sympathetic IChNS, or that an autonomous innervation reaches the IChNS, explaining the presence of numerous NPY vesicles. Additional research is needed to clarify the role of IChNS on retina and macula pathophysiology.

Finally, the close vicinity of nerve fibres with the RPE cells highly suggests that the ChNS also controls RPE cells, which in human express VIP receptor 2, a number of cholinergic receptors (A1, A3, A5, A10, B1) and CGRP receptors [74]. Indeed, autonomous innervation contributes to the maintenance of the outer retinal barrier, as shown for VIP which enhances junction proteins and trans-epithelial resistance in RPE in culture [45], and for NPY involved in Cl^−^ and fluid transport [3].

Overall, the structure and distribution of the ChNS components highlight its role not only in the control of all choroidal vessel types but also in the regulation of immune cells and possibly RPE cell functions (Supplementary Fig. 4).

### MR-induced choroidal neuropathy

Overactivation of the mineralocorticoid signalling contributes to the pathogenesis of several retinal diseases including diabetic retinopathy, choroidal neovascularization and retinopathy of prematurity [5]. In rodent models, MR overactivation, either by chronic aldosterone exposure or by overexpression of the MR (P1.hMR mice), leads to dilated veins in direct contact with Bruch membrane and effacement of the overlying choriocapillaris (“pachyvessel”), irregular pigment distribution in the RPE and subretinal migration of RPE cells [15], which resemble pachychoroid epitheliopathy described in humans [1, 9, 19].

Although MR is expressed in human choroidal endothelial cells, retinal pigment epithelial cells, pericytes and in Schwann cells [74], and regulates the expression of genes involved in pathogenic mechanisms in human RPE cells derived from iPSc [16], the exact mechanism linking overactivation of the mineralocorticoid pathway and pachychoroid phenotype was missing.

Using TEM and SBF-SEM, we show herein typical signs of choroidal neuropathy in P1.hMR mice including myelin abnormalities, accumulation and enlargement of mitochondria and loss of organisation and vacuolization of choroidal nerves. Thus, hMR overexpression in mouse causes a pachychoroid-like phenotype and a choroidal neuropathy. Since, the ChNS regulates all choroidal vessels, macrophages and possibly RPE cell functions, choroidal neuropathy secondary to MR overactivation could be the unifying link between all pathological signs.

The involvement of the mineralocorticoid pathway signalling in the dysfunction of the autonomic nervous system has been demonstrated in other organs. In the heart, MR overactivation favours arrhythmia [49, 58] and aldosterone/renin ratio was associated with heart rate variability (HRV), a surrogate of autonomic dysfunction [36]. In addition, MR antagonists (MRA) were shown to exert beneficial effects on autonomic dysfunction in the context of heart failure [26], suggesting that MR signalling pathway exerts a neurohormonal control.

In CSCR patients, in which choroidal phenotype (vasodilation and leakage) could be attributed to MR overactivation, signs of dysautonomia have also been measured, such as low HRV [7, 70] and abnormal pupillometric response [86]. Moreover, MRA regulates macular choroidal blood flow under exercise-induced hypertension, indicating that MRA acts on the neural control of choroidal blood flow [34].

Whilst autonomic nervous system dysfunction has been observed by various methods in patients with CSCR, these abnormalities have not been linked to possible MR hyper-activation, whereas several studies have shown that MR antagonists can act on the pathological signs of the disease [28, 84]. Furthermore, the link between the MR and the autonomic nervous system in other organs has not been clearly elucidated, and to date, no study has demonstrated structural signs of neuropathy in pathologies associated with MR hyperactivation.

Our results therefore provide new evidence that MR hyperactivation induces a choroidal neuropathy that can explain the pachychoroid phenotype in P1.hMR mice that otherwise show systemic signs of dysautonomia such as arrhythmia [47]. These results allow us to hypothesise that in patients with CSCR, the systemic signs of dysautonomia could be linked to the choroidal phenotype through a choroidal neuropathy, possibly induced by MR hyperactivation. This hypothesis will have to be verified in humans by functional methods that remain to be developed.

### Molecular mechanisms linking hMR overexpression and pachychoroid pigment epitheliopathy phenotype

Since MR acts as a transcription factor, we have performed a pantranscriptomic differential analysis of the RPE/choroid complex between 5-month-old P1.hMR mice and their WT littermates. Pathways related to vasculature development, hypoxia, epithelial cell differentiation/apoptosis, epithelial mesenchymal transition and inflammation were deregulated supporting the observed phenotype and highlighting potential downstream molecular targets. Amongst the differentially regulated genes in transgenic animals, several are known as corticoids-regulated genes involved in neuropathology and pathology of epithelial tissues. Lipocalin 2 (NGAL encoded by *Lcn2* gene), a known target gene of aldosterone/ MR activation via NF-κB pathway [14, 51, 72], is a biomarker of neuro-inflammation and inhibits remyelination [2, 35, 37]. Ras-Related Dexamethasone Induced 1 (*rdas1/dexras1*), upregulated by corticoid [13] induces oligodendrocyte dedifferentiation and myelin injury [43, 81], whilst its deletion promotes neuroprotection.

Aquaporin 3 (*Aqp3*), induced by MR signalling [46], is increased in human RPE cells in response to hypoxia [39]. Related to this process, inhibitor of DNA binding 2 (*Id2*) is downregulated by 1.5-fold in P1.hMR mice. This gene is downregulated by aldosterone in the heart [41] and it protects against hypoxia in the RPE [31]. ERBB Receptor Feedback Inhibitor 1 (*Errfi1*), upregulated by glucocorticoids and in P1.hMR promotes the apoptosis of epithelial cells [82].

Genes related to neuro-inflammation processes were also found upregulated in transgenic P1.hMR mice including *Sphk1*, *Ccl2*, *S100a8*, *S100a9* and *Socs3*. The sphingosine kinase 1 (*Sphk1*) is linked to NF-κB signalling, promoting cytokines production by activated microglia and participating in neuronal injury and neuro-inflammation after ischaemia [55, 69]. The chemokine Ccl2 is involved in neuropathic pain, inflammation, and in neuro-glia interaction during pathogenesis [85]. *S100a8* and *S100a9* upregulation is representative of a pro-inflammatory state in injured peripheral nerves and induces microglia activation [17, 80]. Finally, several genes related to neuronal function are downregulated in P1.hMR mice, such as genes encoding for rabphilin 3a (*Rph3a*), secretogranin (*Scg2*) and Kinesin Family Member 5A (*Kif5a*), respectively, important for synaptic transmission [67, 71, 87], abundantly expressed by trigeminal neurons innervating the eye [63, 64] and a crucial regulator of neurodegeneration [65]. Whilst an important proportion of neuronal RNA is missing due to the lack of neuronal bodies in the rodent choroid, the regulated genes/pathways reflect potential underlying mechanisms linking chorioretinopathy with choroidal neuropathy.

### Potential application for clinical images analysis

In patients with pachychoroid and central serous choroidopathy, typical perivascular densities and hyperreflective structures have been recognised [24], but until now they have not been connected to neural structures. Hyperreflective dots have been identified as potential inflammatory cells, but such dots vary with time and activity of the disease [52], which is very different from the perivascular hyperreflective bands that remain stable over time. Whilst the present work better characterised the ChNS using either histology or immunohistochemistry, recognised its density around vessels, its organisation, the large size of certain fibres, the presence of ganglion cells and its proximity with the choriocapillaris, it seems very plausible that the ChNS could be imaged by SD-OCT or infrared images when the RPE is damaged. In case of choroidal neuropathy, the morphological changes of nerves, shown in the P1.hMR mouse model, could translate to the abnormal perivascular structures observed in CSCR cases. Interestingly, genetic variants in VIP receptor 2 (VIPR2), have been associated with choroidal thickness and with pachychoroid-associated CSCR [40], reinforcing the idea of a potential causal link between neural pathology and pachychoroid. The association of this choroidal phenotype with evidence of systemic dysautonomia reinforces the hypothesis that the pachychoroid may result from a choroidal neuropathy.

We recognise some weakness in this study, such as the absence of SD-OCT images in the mouse model and strict anatomical-clinical correlation studies. The resolution of SD-OCT in mouse does not allow to acquire potential ChNS images and human donor eyes with CSCR are, to our knowledge, not available in eye banks. To further progress in correlative studies, we have generated a transgenic rat model that overexpresses hMR and has larger eyes, and that is currently under investigation. The choroid is a difficult tissue to study due to the adherence of the RPE, the presence of pigments and its deep location, explaining the scarcity of data available on the histological description of human choroidal neural system.

In summary, we have described the distribution of ChNS and its close anatomical relationship with all choroidal vessels, immune cells and RPE in the human and rodent choroid, suggesting a central role of ChNS not only in the regulation of choroidal blood flow, but also in the function of the innate immune system and of the RPE. The abnormal diameter of choroidal vessels and RPE changes observed in P1.hMR mice could be a consequence of MR-induced deregulation of the ChNS. The morphological changes in the choroidal nerves observed in P1.hMR mice could be translated to the abnormal hyperreflective perivascular structures present on OCT and ICG of patients with pachychoroid spectrum diseases. The hypothesis that MR-mediated choroidal neuropathy could be the link between corticoids and pachychoroid, and the use of imaging markers of choroidal neuropathy in the diagnosis and treatment of choroidal diseases merits further clinical investigations.

### Supplementary Information

Below is the link to the electronic supplementary material.

Supplementary file1 (PDF 1018 KB)

Supplementary file2 (MPG 26000 KB)

Supplementary file3 (XLSX 2422 KB)

## Data Availability

RNAseq data are avilable in supplementary Table 3 and all other raw data are available upon reasonable request.
