# Supplementary material for: The choroidal nervous system: a link between mineralocorticoid receptor and pachychoroid
Source: Acta Neuropathol. 2023 Sep 8;146(5):747–66. doi: 10.1007/s00401-023-02628-3 (PMC10564818; doi:10.1007/s00401-023-02628-3)
Supplement: Supplementary file 1 — Supplementary file1 (PDF 1018 KB) [file 401_2023_2628_MOESM1_ESM.pdf]

Supplementary online information for

The choroidal nervous system:
A link between mineralocorticoid receptor and pachychoroid

Bastien Leclercq[1], Allon Weiner[2], Marta Zola[1,3,4], Dan Mejlacowicz[1], Patricia Lassiaz[1], Laurent Jonet[1], Emmanuelle Gélizé[1], Julie Perrot[5], Say Viengchareun[5], Min Zhao[1], Francine Behar-Cohen[1,3,4].

[1] Centre de Recherche des Cordeliers, Inserm, Université Paris Cité, Sorbonne Université, Physiopathology of ocular diseases : Therapeutic innovations, Paris, France

[2] Sorbonne Université, Inserm, Centre d'Immunologie et des Maladies Infectieuses, Cimi-Paris, Paris, France.

[3] Ophthalmopole Cochin University Hospital, Assistance Publique-Hôpitaux de Paris, France

[4] Hopital Foch, Suresnes, France

[5] Université Paris-Saclay, Inserm, Physiologie et Physiopathologie Endocriniennes, 94276, Le Kremlin-Bicêtre, France

Corresponding author

Francine Behar-Cohen, 15 rue de l'Ecole de Médecine 75006 Paris, France, francine.behar@gmail.com

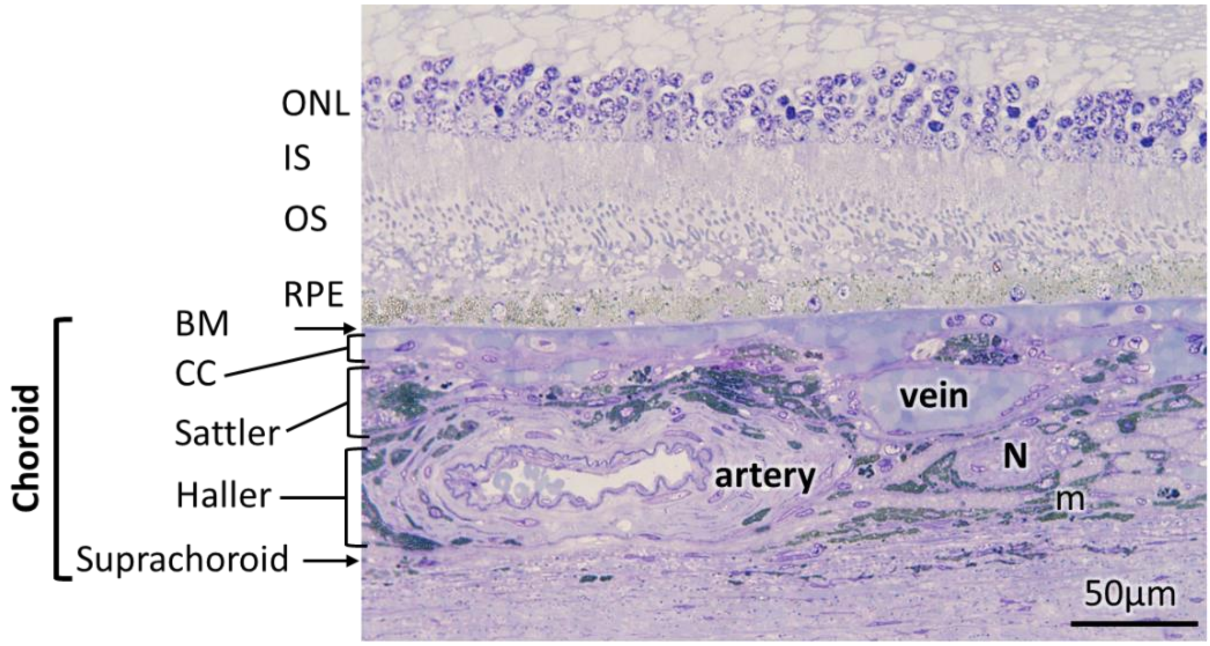

Supplementary Figure 1: Histology of human choroid in the macula. Choroid and outer retina paraffin section in the macula region of a human eye. ONL: outer nuclear layer ; IS: photoreceptor inner segments ; OS: photoreceptor outer segments ; RPE: retinal pigment epithelium ; BM: bruch's membrane ; CC : choriocapillaries ; N: choroidal nerve ; m: melanocyte.

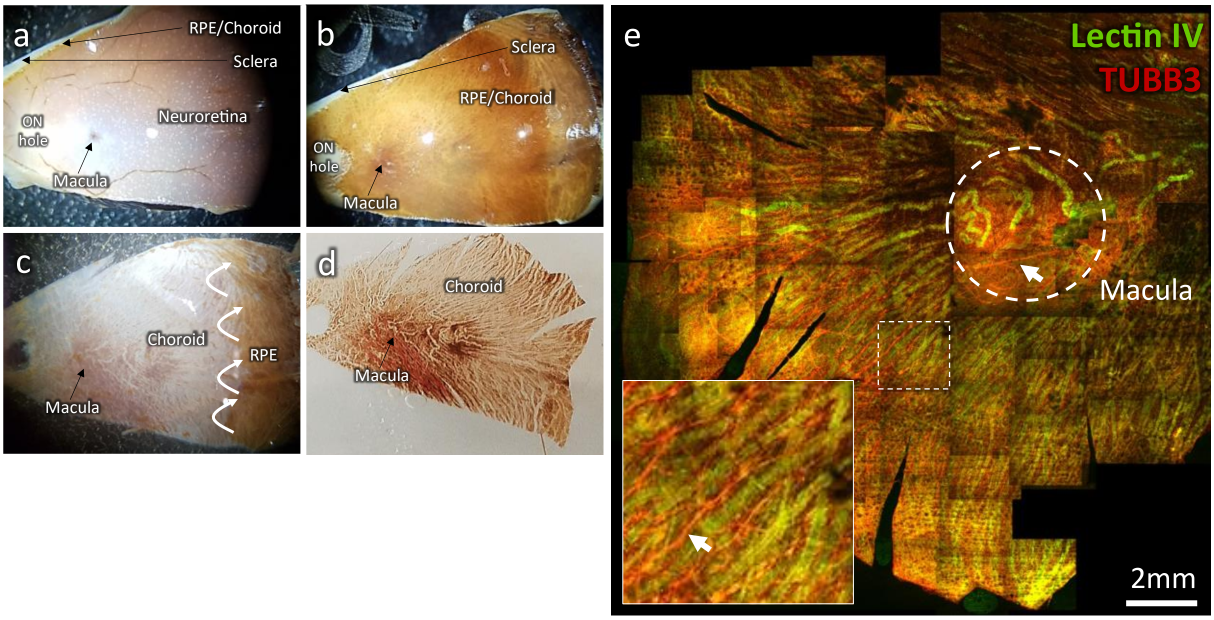

Supplementary Figure 2: Dissection of human choroid and flat-mounted visualization by immunostaining of human choroidal nervous system. (**a-d**) Show the different steps of tissue dissection. The retina is removed (**a-b**), then the RPE is delicately detached from the choroid using a paintbrush (**C**, arrows show direction of removing) and finally the choroid is separated from the sclera (**d**). (**e**) Mosaic acquisition of human choroidal immunostaining of Lectin IV and TUBB3 allowing visualisation of choroidal vasculature (green) and choroidal nerves (red, white arrows), notably within the macular zone.

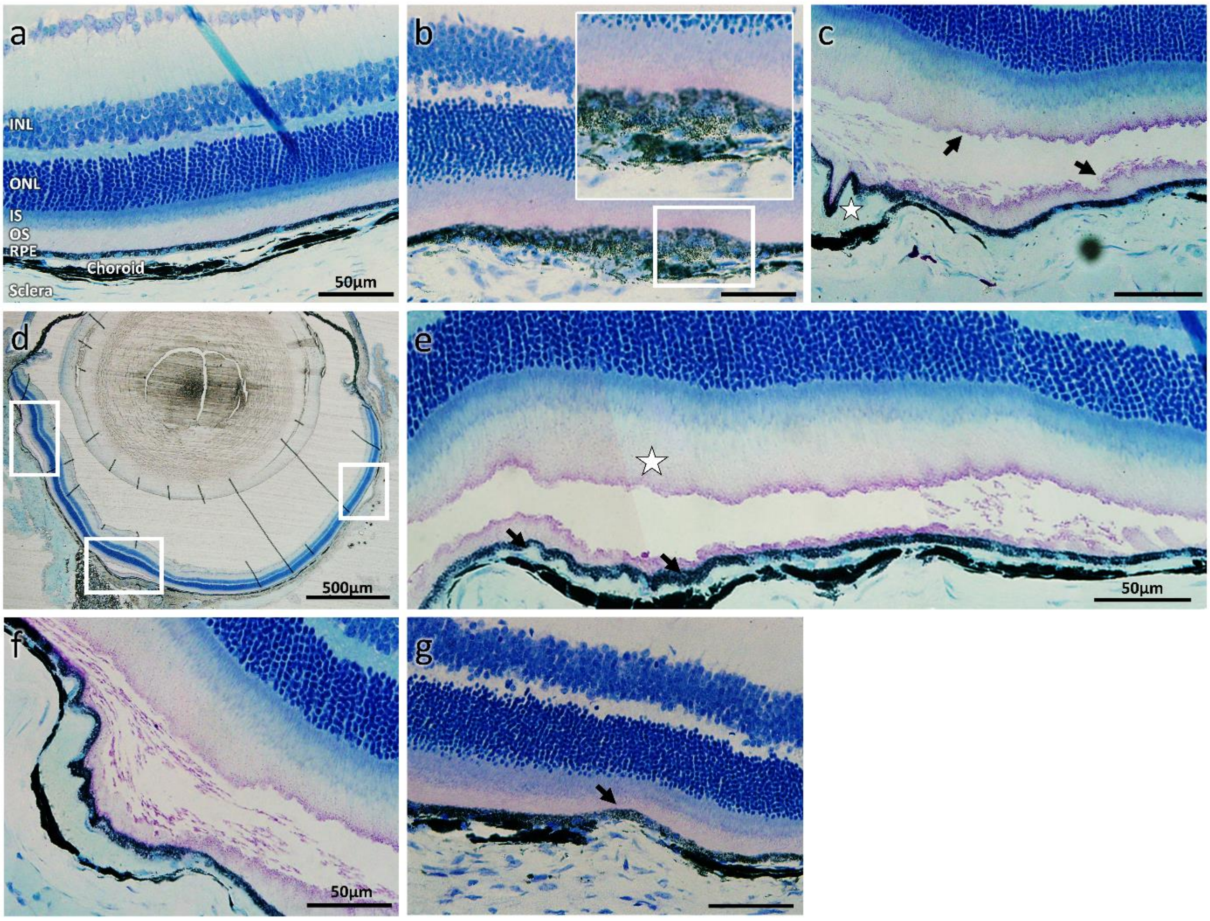

Supplementary Figure 3: Triamcinolone treatment leads to a worsening of P1.hMR mice chorioretinopathy. Transversal eyes sections (embedded in HistoResin and stained with toluidin blue) of P1.hMR mice and their WT littermates which received subconjunctival Triamcinolone acetonide (TA) injection. Historesin sections offer a limiting anatomical resolution compared to semithin section, which explains the RPE anatomical differences when comparing with figure 5. (**a**) TA injection in WT mice do not induce any significant changes in the choroid nor in the retina. (**b-g**) P1.hMR mice which received TA injection show a worsening of the chorioretinopathy. This includes abnormal RPE proliferation and pigment dispersion (**b, e** arrows, **g** arrow), elongation of photoreceptor segments and local retinal detachments (**c** arrows**, d**, **e** star, **f**), large choroidal vessel dilation and RPE fold (**c** star, **e** arrows, **g** arrow). Overall, TA injection is worsening the chorioretinopathy previously observed in P1.hMR mice. INL : inner nuclear layer; ONL: outer nuclear layer; IS : photoreceptor inner segments; OS: photoreceptor outer segments; RPE: retinal pigment epithelium.

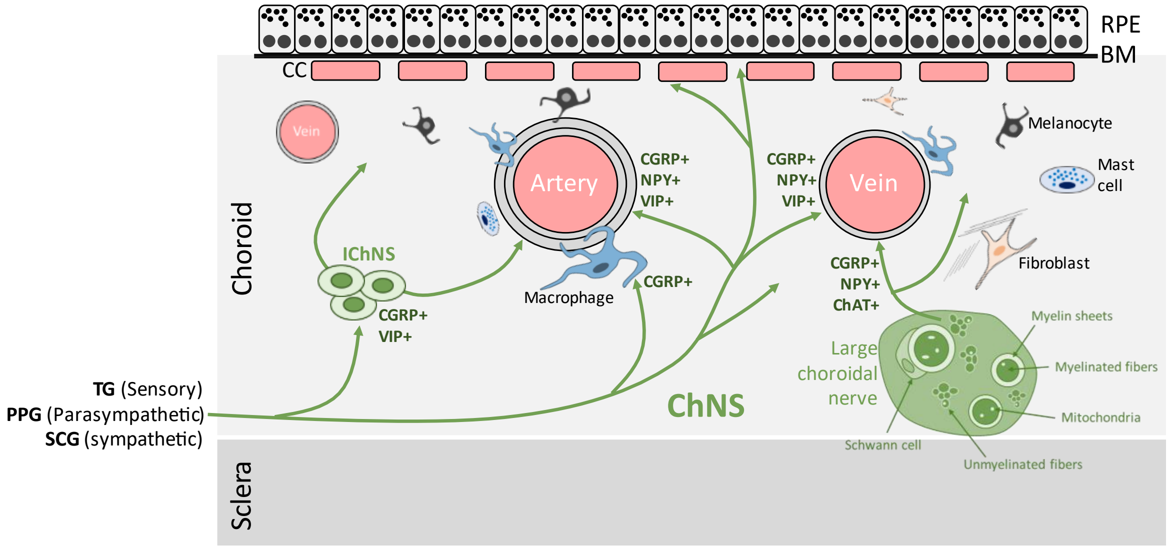

Supplementary Figure 4: Schematic representation of choroidal innervation. Fibers from trigeminal ganglion (TG) pterygopalatine ganglions (PPG) and superior cervical ganglion (SCG) innervate the choroid, including arteries, veins and choriocapillaries (CC). Autonomous (NPY+/VIP+/ChAT+) and sensory (CGRP+) fibers are found around the choroidal arteries and veins. CGRP+ fibers are also found contacting choroidal macrophages. CGRP+, NPY+ and ChAT+ bifurcations from ciliary nerves (large choroidal nerves) are entering the choroid as well. In foveate animals like humans, intrinsic choroidal neurons (IChNS) also innervate the choroid and receive input from autonomous/sensory ganglions. ChNS : choroidal nervous system RPE: retinal pigment epithelium, BM: Bruch's membrane.

Supplementary file 1: Video animation of the 3D reconstruction of the SBF-SEM volume presented in figure 3. The myelin segmentation is in purple, the choroidal nerve fiber in magenta and the choriocapillaries in red. The reconstruction reveals the ultrastructural organization of the choroidal innervation, with a clear innervation of large vessels. Video - WT mouse 3D segmentation file.

Supplementary file 2: Raw and processed transcriptomic data obtained from bulk RNAseq of RPE/choroid complex of P1.hMR mice and their WT littermates. The data discussed in this publication have been deposited in NCBI's Gene Expression Omnibus and are accessible through GEO Series accession number GSE233432 (https://www.ncbi.nlm.nih.gov/geo/query/acc.cgi?acc=GSE233432)
